# ﻿Two new species of *Dolichurus* Latreille (Hymenoptera, Ampulicidae) from China, with a key to species of the genus

**DOI:** 10.3897/zookeys.1254.161007

**Published:** 2025-10-03

**Authors:** Xuemei Bai, Li Ma, Guowen Tang, Qiang Li

**Affiliations:** 1 Department of Entomology, College of Plant Protection, Yunnan Agricultural University, Kunming, Yunnan, 650201, China Yunnan Agricultural University Kunming China

**Keywords:** Cockroach wasps, Dolichurinae, *
Dolichurus
*, key, sphecid wasps, taxonomy

## Abstract

Two new species of *Dolichurus* Latreille, 1809, namely *D. albipedigerus* Bai & Li, **sp. nov.** and *D. nigrilamellatus* Bai & Li, **sp. nov.** are described from China (Yunnan, Guangdong and Jiangxi Provinces). *Dolichurus albipedigerus* Bai & Li, **sp. nov.** is similar to *D. apiciornatus* Tsuneki, 1977, but differs in having the mandible ivory white medially, the clypeus with a weak median carina, and the body with ivory-white spots. *Dolichurus nigrilamellatus* Bai & Li, **sp. nov.** is similar to *D. aposanus*Tsuneki et al., 1992, but differs in having the frons with dense rugae converging toward the midline above the lamella, the sternaulus conspicuous, and the posterior margin of T1-T3 without tiny puncture-rows medially. Additionally, a key to the Chinese species of *Dolichurus* is provided.

## ﻿Introduction

Wasps of the genus *Dolichurus* Latreille, 1809 are small sphecids that use cockroaches as prey for larvae, commonly called cockroach wasps. *Dolichurus* belongs to Ampulicidae, Dolichurini, and is the largest genus in the tribe Dolichurini. Latreille (1809) erected *Dolichurus* without including any species, and later (1810) designated *D. corniculus* (Spinola, 1808) as the type species of the genus (Latreille 1809, 1810). At present, *Dolichurus* comprises 54 species and two subspecies worldwide, mainly found in the Oriental, Palearctic and Ethiopian regions, with fewer species occurring in the Nearctic, Neotropical and Australo-Papuan regions. Fifteen species occur in the Palearctic region, 29 species and one subspecies in the Oriental, two species in the Nearctic, 13 species and one subspecies in the Ethiopian, three species in the Neotropical, and four species in the Australo-Papuan; eight species are shared between the Palearctic and Oriental, two species are shared between the Palearctic and Ethiopian, and one species is shared between the Nearctic and Neotropical regions (F. Smith 1869, Arnold 1928, Arnold 1952, Tsuneki 1967, Nagy 1971, Tsuneki 1972, Tsuneki 1977, Krombein 1979, Tsuneki 1992, Tsuneki et al. 1992, Ohl 2011, Girish Kumar and Sheikh 2018, Anagha et al. 2020, Pulawski 2025). Currently, 12 species are known in China (Pu 1986, Wu et al. 2003, Hua 2006, Pulawski 2025).

In the distant past, China was influenced by geologic events such as continental drift and the accelerated uplift of the Tibetan Plateau. These processes have not only shaped current distribution patterns, unique landscapes and landforms, and geographical barriers, but also promoted rapid differentiation of organisms, thereby enriching biodiversity. China is situated in the southeastern part of Eurasia, straddling the Oriental and Palearctic regions. Moreover, China had complex and diverse landform patterns and climatic environments, which provided a rich variety of habitat types for insects, forming both widespread and endemic species. Rich biodiversity is conducive to the multiplication and thriving of cockroach wasps (Almeida et al. 2012; Da Silva and Noll 2015; Trewick 2017).

In this study, two new species, *D. albipedigerus* Bai & Li, sp. nov. and *D. nigrilamellatus* Bai & Li, sp. nov. were discovered and described in detail, including a differential diagnosis with their nearest species, a key to known species in China, and photographs of the two new species.

## ﻿Material and methods

The specimens examined are deposited in Yunnan Agricultural University, Kunming, China (YNAU). Specimens were photographed using a stereomicroscope (Keyence VHX-S5500E) equipped with a digital microscopic system. Plates were processed with Adobe Photoshop® 2020 software. For the terminology, we mainly follow Bohart and Menke (1976). The abbreviations are as follows:

**HW** head width (dorsal view, maximum)

**HL** head length (frontal view, distance from vertex to margin of clypeus in the middle)

**POD** post-ocellar distance (dorsal view, distance between inner margins of hind ocellus)

**OD** ocellus distance (dorsal view, minimum distance between inner and outer margin of ocellus)

**OOD** ocellocular distance (dorsal view, distance between outer margin of hind ocellus and nearest inner orbit)

**IODv** minimum interocular distance in vertex (frontal view)

**IODc** minimum interocular distance in clypeus (frontal view)

**A** antenna

**aW** antennal apical width

**bW** antennal basal width

**T** gastral tergum (dorsal view)

**S** gastral sternum (ventral view)

**LT** maximum length of gastral tergum (dorsal view)

**WT** maximum width of gastral tergum (dorsal view)

## ﻿Result

### ﻿ Dolichurus

Taxon classificationAnimaliaHymenopteraAmpulicidae

Latreille, 1809

3AE18C0C-2141-5AD8-940C-D401EE51E03F

Dolichurus Latreille, 1809: 387. Type species: Pompilus corniculus Spinola, 1808, designated by Latreille, 1810: 438.
Thyreosphex Ashmead, 1904: 282. Type species: Thyreosphex stantoni Ashmead, 1904, by monotypy.

#### Diagnosis.

Medium to small wasps, 5–13 mm. Black, terminal gastral segments sometimes red; whitish marks on mandible, clypeus, frontal platform, pronotal tubercle, tegula, and leg. Inner margin of mandible with teeth. Clypeus median carina often present. Antenna filiform, slender and long; 12 segments in female, 13 segments in male; antennal sockets nearly contiguous, covered by U-shaped lamella. Frontal line usually indicated, but weak and intermittent; vertex arched above eyes; maxillary palpi slender, labial palpi short and stout; thorax covered with silvery pubescence, pronotal collar shorter than scutum; scutum notauli conspicuous and nearly complete, admedian line absent, parapsidal line present, scutellum with a transverse furrow anteriorly; mesopleuron with omaulus, anteroventral remnant of episternal sulcus; acetabular carina incomplete; sternaulus and scrobal sulcus conspicuous to inconspicuous; propodeum with U-shaped enclosure, multicarinate to areolate, propodeum setae dense and long; petiole and pygidium absent. T1 anterior inclination with shallow oval depression, usually impunctate, S1 appears humped basally and medially, and forms lamella apically, S2 with deep groove at base. Forewing with three submarginal cells, media diverging after cu-a; hindwing jugal lobe present but small, media diverging before cu-a (Bohart and Menke 1976).

#### Biology.

Larve of *D. corniculus* (Spinola, 1807) have been described by Maneval (1939) and Evans (1959). Nests of *Dolichurus* are sealed with leaves and grass debris, primarily in stalks and rock crevices, or buried in holes 7–8 cm deep in the ground (Ferton 1895). *Dolichurus greenei* Rohwer, 1916, *D. corniculus* (Spinola, 1807), and *D. turanicus* Gussakovskij, 1940 prey on species of Ectobiidae (Grandi 1931, Krombein 1955); *D. corniculus* (Spinola, 1807) prey on species of Blattidae (Handlirsch 1889); and *D. haemorrhous* A. Costa, 1886 prey on species of Blattellidae (Adlerz 1904).

##### ﻿Key to the species of *Dolichurus* Latreille, 1809 from China

(Females are unknown for *D. dromedarius* Nagy, 1971; *D. maculicollis* Tsuneki, 1967; *D. ombrodes* Nagy, 1971; and *D. shirozui* Tsuneki, 1967. Males are unknown for *D. alorus* Nagy, 1971; *D. apiciornatus* Tsuneki, 1977; *D. formosanus* Tsuneki, 1967; *D. leioceps* Strand, 1913; *D. nigrilamellatus* Bai & Li, sp. nov.; and *D. pempuchiensis* Tsuneki, 1972.)

**Table:** 

1	Female (antenna 12 segments; gaster with six segments visible; inner margin of mandible with 2–3 teeth)	**2**
–	Male (antenna 13 segments; gaster with three segments visible; inner margin of mandible unidentate subapically)	**11**
2	Pronotal collar with transverse carina conspicuous anteriorly	**3**
–	Pronotal collar without transverse carina anteriorly	**6**
3	Clypeus without median carina (China; East Asia; Southeast Asia)	***D. leioceps* Strand, 1913**
–	Clypeus with median carina, carina confined to basal half	**4**
4	Mesopleuron sternaulus conspicuous (China: Taiwan; East Asia; India; Japan; Malaysia; Southeast Asia; Thailand; Vietnam)	***D. amamiensis* Tsuneki & Iida, 1964**
–	Mesopleuron sternaulus inconspicuous or lacking	**5**
5	Lamella nearly as long as wide (China: Taiwan)	***D. pempuchiensis* Tsuneki, 1972**
–	Lamella relatively longer (China: Taiwan)	***D. formosanus* Tsuneki, 1967**
6	Clypeus with ivory-white spot	**7**
–	Clypeus black	**8**
7	Anterior part of clypeus with small transverse ivory-white spot; mandible ferruginous; lateral surface of propodeum with weak, short carinae (China: Taiwan; East Asia; Laos; Southeast Asia)	***D. apiciornatus* Tsuneki, 1977**
–	Anterior part of clypeus with large ivory-white spot; mandible ivory white medially (Fig. 1F); lateral surface of propodeum with conspicuous, longitudinal carinae (Fig. 1D) (China: Guangdong, Jiangxi, Yunnan)	***D. albipedigerus* Bai & Li, sp. nov.**
8	Clypeus without median carina	**9**
–	Clypeus with conspicuous median carina	**10**
9	Anterior inclination of pronotum finely and densely punctate; mesopleuron densely punctate (China: Taiwan; Southeast Asia)	***D. alorus* Nagy, 1971**
–	Anterior inclination of pronotum with close transverse wrinkles; mesopleuron with small mesh (China; India; Southeast Asia; Sri Lanka)	***D. aridulus* Krombein, 1979**
10	Mesopleuron sternaulus conspicuous (Fig. 3C); frons with rugae converging toward midline, mixed with few large punctures; lamella length almost equal to width (Fig. 3D) (China: Guangdong, Jiangxi)	***D. nigrilamellatus* Bai & Li, sp. nov.**
–	Mesopleuron sternaulus inconspicuous; frons coarsely reticulate; lamella markedly broader than long (China: Taiwan; East Asia; Southeast Asia)	***D. abbreviatus* Strand, 1913**
11	Pronotal collar with transverse carina conspicuous anteriorly (China: Taiwan; East Asia; India; Japan; Malaysia; Southeast Asia; Thailand; Vietnam)	***D. amamiensis* Tsuneki & Iida, 1964**
–	Pronotal collar without transverse carina anteriorly	**12**
12	Clypeus ivory white except midline	**13**
–	Clypeus black	**14**
13	Mandible ferruginous; tegula yellowish brown, hypoepimeral area black; leg ferruginous; T1 and T2 scattered with tiny punctures (China: Taiwan; Laos; Malaysia; Southeast Asia)	***D. ombrodes* Nagy, 1971**
–	Mandible ivory white medially (Fig. 2C); anterior half of tegula and hypoepimeral area ivory white (Fig. 2C, E); leg with ivory-white spots (Fig. 2A); posterior margin of T1 and T2 with tiny punctures, remainder smooth (Fig. 2H) (China: Guangdong)	***D. albipedigerus* Bai & Li, sp. nov.**
14	Lamella black (China: Taiwan; East Asia; Southeast Asia)	***D. abbreviatus* Strand, 1913**
–	Apical margin of lamella ivory white	**15**
15	Pronotal tubercle black (China: Taiwan; Southeast Asia)	***D. dromedarius* Nagy, 1971**
–	Pronotal tubercle ivory white	**16**
16	Mesopleuron smooth, scattered with tiny punctures (China: Taiwan; Southeast Asia)	***D. maculicollis* Tsuneki, 1967**
–	Mesopleuron with dense rugae or punctures	**17**
17	Mesopleuron with dense rugae, forming small mesh; gaster with large punctures (China; India; Southeast Asia; Sri Lanka)	***D. aridulus* Krombein, 1979**
–	Mesopleuron with dense punctures posteriorly, punctures gradually finer and sparser anteriorly; gaster with midsized punctures, segmental punctures slightly sparser posteriorly (China: Taiwan; Southeast Asia)	***D. shirozui* Tsuneki, 1967**

### ﻿ Dolichurus albipedigerus

Taxon classificationAnimaliaHymenopteraAmpulicidae

Bai & Li sp. nov.

DC2E2FDC-0001-5ED5-A3DD-BC7DF09EEF2A

https://zoobank.org/37A10406-A8E5-46AF-BE74-9739ED3F6EDF

Figs 1A–G, 2A–K

#### Type material.

***Holotype***. China • ♀; Yunnan Province, Xishuangbanna City, Tropical Botanical Garden Rubber Plantation; 22°8′3.48′′N, 100°43′5.16′′E; 24.IV−31.V.2019, collected by Qiang Li; malaise trap. ***Paratypes***. China • 1♂; Guangdong Province, Shaoguan City, Nanling Nature Reserve, Chebaling; 24°42′50.15′′N, 114°15′43.59′′E; 464 m a.s.l.; 01.V−16.VI.2022, collected by Institute of Zoology, Guangdong Academy of Sciences; malaise trap. China • 1♀; Yunnan Province, Xishuangbanna City, Tropical Botanical Garden rainforest 2; 21°40′N, 101°24′E; 11.IV−16.V.2021, collected by Li Ma; malaise trap. China • 1♀; Guangdong Province, Shaoguan City, Nanling Nature Reserve, Chebaling; 24°42′45.34′′N, 114°15′35.94′′E; 449 m a.s.l.; 13.VIII−23.IX.2020, collected by Institute of Zoology, Guangdong Academy of Sciences; malaise trap. China • 1♀; Guangdong Province, Shaoguan City, Nanling Nature Reserve, Scale Frame; 24°49′59.44′′N, 112°47′50.23′′E; 360 m a.s.l.; 23−27.V.2023, collected by Institute of Zoology, Guangdong Academy of Sciences; malaise trap. China • 1♀; China, Guangdong Province, Shaoguan City, Nanling Nature Reserve, Forward Station; 24°26′56.01′′N, 113°7′54.28′′E; 782 m a.s.l.; 23.VII−7.VIII.2021, collected by Institute of Zoology, Guangdong Academy of Sciences; malaise trap. China • 1♀; Jiangxi Province, Longnan City, Jiulian Nature Reserve; 24°32′17.99′′N, 114°27′52.37′′E; 620 m a.s.l.; 23.VIII−4.IX.2020, collected by Institute of Zoology, Guangdong Academy of Sciences; malaise trap.

#### Diagnosis.

The new species is similar to *D. apiciornatus* Tsuneki, 1977, but differs from it and other congeners by the following characters (characters of *D. apiciornatus* in parentheses): 1) mandible ivory white medially (mandible ferruginous); 2) OOD: POD = 7: 5 (OOD: POD = 7: 6); 3) IODv: IODc = 32: 45 (IODv: IODc = 30: 40); 4) clypeus with weak median carina, anterior part with large ivory-white spot (clypeus without median carina, anterior part with small ivory-white spot); 5) lateral surface of propodeum with conspicuous, longitudinal carinae (lateral surface of propodeum with weak, short carinae anteriorly and posteriorly); and 6) body with ivory-white spots (body black).

#### Description.

**Figure 1.**
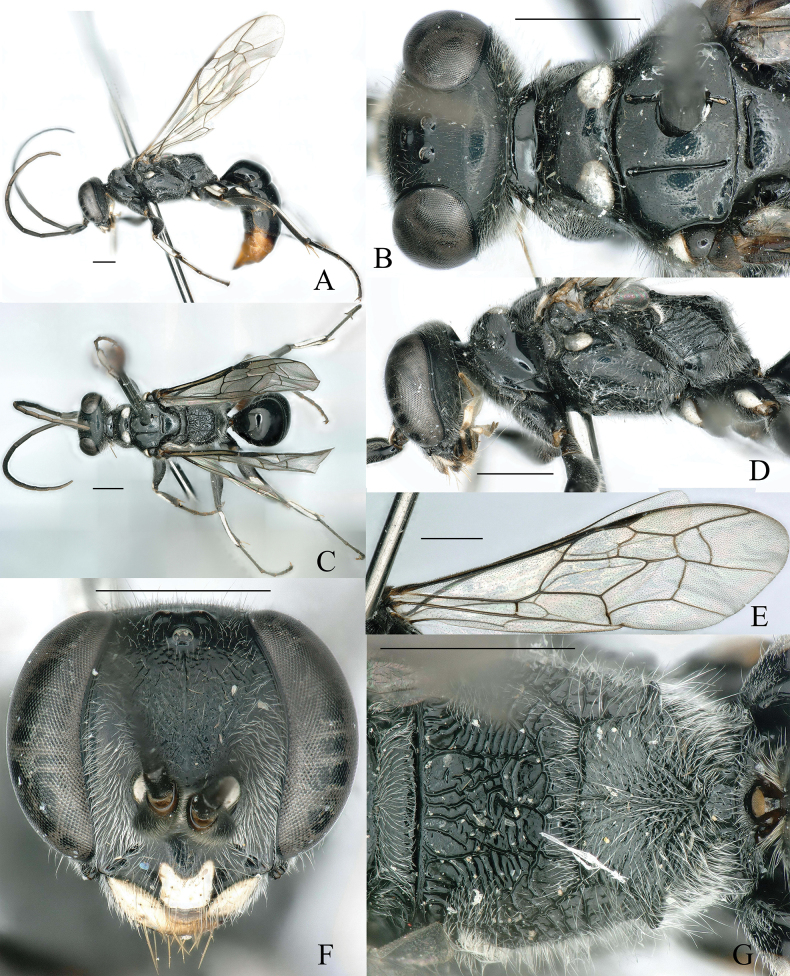
A–G. *Dolichurus albipedigerus* Bai & Li, sp. nov., holotype, ♀. A. Habitus, lateral view; B. Head, pronotum, scutum, and scutellum, dorsal view; C. Habitus, dorsal view; D. Head and thorax, lateral view; E. Eing; F. Head, frontal view; G. Propodeum, dorsal and posterior view. Scale bars: 1 mm.

**Female**. Body length 10.0−11.3 mm. Black. Nearly entire apical half of lamella ivory white, middle boundary M-shaped; anterior part of clypeus with ivory-white spot, apical lobe transparent yellow. The following ivory white: mandible medially, palpi largely, pronotal tubercle, anterior half of tegula, hypoepimeral area; remaining palpi light brown. The following with ivory-white marks: fore femora apically, outside of mid and hind coxae, tibiae and hind femora. Fore and mid tibiae spurs white, hind tibiae spurs light brown; remainder brown. Wing hyaline and iridescent, veins and stigma light brown (Fig. 1E). Gastral segments IV−VI reddish brown. Vestiture silvery pubescence; most of frons and vertex with silvery, erect pubescence; side of lamella and frons below lamella with appressed silvery pubescence; mandible with yellow, erect macrochaetae. Gaster smooth, practically impunctate (Fig. 1A, B).

***Head***. In front view, nearly subcircular in outline, HW: HL = 90: 85. Mandible broad and blunt, inner margin tridentate. Clypeus smooth, convex medially, with weak median carina in basal half, apical lobe subtruncate, base width: middle width: apical width = 15: 45: 10 (Fig. 1F). Antennae 12 segments, scape with weak ventral carina. Relative length of antennal segments I: II: III: IV: V: VI: VII: VIII: IX: X: XI: XII = 26: 9: 37: 31: 32: 30: 26: 23: 20: 20: 19: 17. A3/aW = 5.3, A12/bW = 4.3. Lamella broadened and flat, length: width = 12: 27. Frontal line complete, nearly reaching midocellus, and slightly concave at base of lamella. Frons with weak wrinkles above lamella, mixed with tiny punctures, side of frons with tiny punctures. Inner margins of eyes slightly convergent above, IODv: IODc = 32: 45 (Fig. 1F). POD: OD: OOD = 5: 6: 7, side of ocelli scattered with tiny punctures. In dorsal view, vertex shiny, scattered with tiny punctures, slightly arched above eyes (Fig. 1B). In lateral view, eyes: gena = 29: 12 (Fig. 1D).

***Thorax***. Pronotum smooth, scattered with tiny punctures, without transverse carina anteriorly, small punctures confluent in several transverse puncture-rows anteriorly, pronotal tubercle slightly raised, hardly projects over level of scutum (in lateral view), median impression shallow (Fig. 1B, D). Scutum and scutellum shiny, scattered with tiny punctures (Fig. 1B). Mesopleuron smooth, scattered with tiny punctures, scrobal sulcus inconspicuously depressed, sternaulus conspicuous (Fig. 1D). Metanotum scattered with punctures (Fig. 1G). Dorsal surface of propodeum with six longitudinal carinae, including two longitudinal carinae, two oblique carinae on either side, and two oblique, longitudinal carinae located further out to sides. Posterior surface of propodeum with radiating reticulation, lateral margin with blunt tooth halfway from dorsum (Fig. 1G). In lateral view, dorsal and posterior surfaces forming obtuse angle, lateral surface of propodeum with conspicuous, longitudinal carinae (Fig. 1D). Ventral surface of tarsi with 2 rows of parallel spines (Fig. 1A, C).

***Gaster***. Six segments visible. LT1: WT1 = 37: 66. Terga smooth. Lateral side of T1 with conspicuous, longitudinal carinae. Sterna I-VI with small to midsized punctures (Fig. 1A, C).

**Figure 2.**
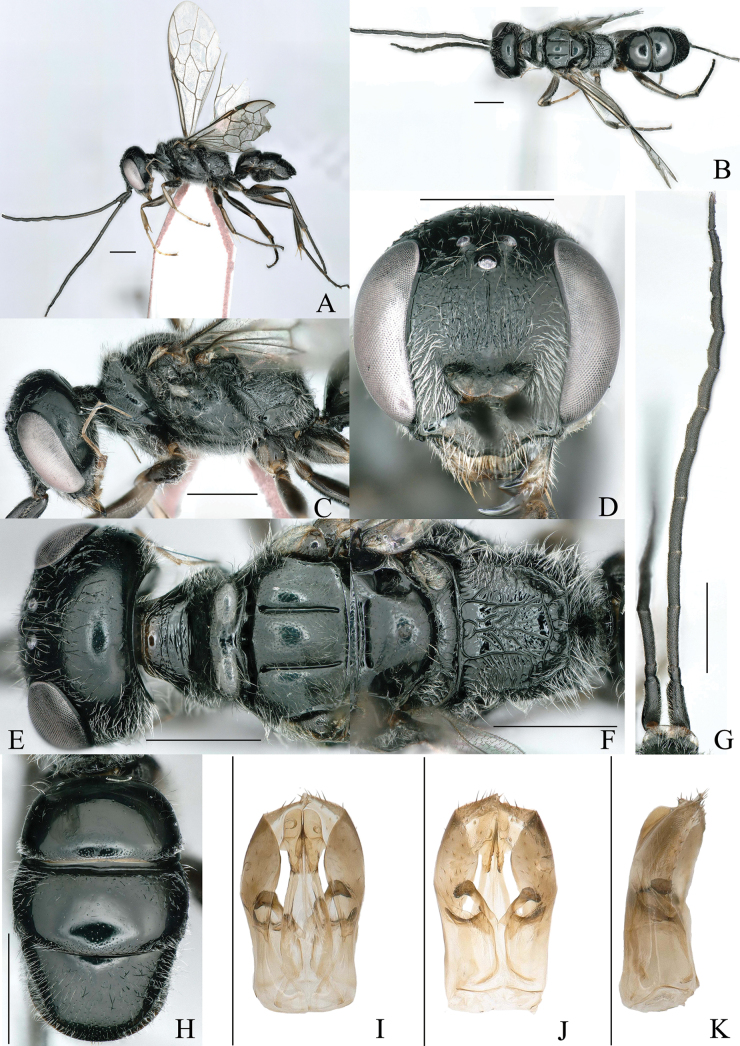
A–K. *Dolichurus albipedigerus* Bai & Li, sp. nov., paratypes, ♂. A. Habitus, lateral view; B. Habitus, dorsal view; C. Head and thorax, lateral view; D. Head, frontal view; E. Head, pronotum, and scutum, dorsal view; F. Scutellum and propodeum, dorsal view; G. Antennae; H. Gaster, dorsal view; I. Genitalia, dorsal view; J. Genitalia, ventral view; K. Genitalia, lateral view. Scale bars: 1 mm.

**Male**. Similar to female, but body smaller, body length 7.0 mm (Fig. 2A, B). Head in front view, HW: HL =83: 77. Most of frons, vertex, and gena with silvery, erect long setae. Mandible sharper and slenderer than that of female, inner margin unidentate subapically. Clypeus ivory white except median line and base, median carina extending to anterior margin, base width: middle width: apical width = 15: 44: 11 (Fig. 2D). Antennae 13 segments, scape ventral carina stronger than female. Relative length of antennal segments I: II: III: IV: V: VI; VII: VIII: IX: X: XI: XII: XIII= 27: 7: 28: 26: 26: 23: 21: 20: 17: 11: 11: 10: 12. A3/aW = 5.6, A12/bW = 4, A6-11 slightly swollen in middle on ventral surface, swelling bearing conspicuous, erect seta (Fig. 2G). Inner margins of eyes slightly convergent above, IODv: IODc = 42: 45 (Fig. 2D). POD: OD: OOD = 8: 6: 10 (Fig. 2D, E). In dorsal view, arched above eyes, stronger than female (Fig. 2E). In lateral view, eyes: gena = 23: 23, palpi light brown (Fig. 2C). Thorax covered with silvery long setae (Fig. 2C, E, F). Pronotum narrower than female (Fig. 2C, E). Dorsal surface of propodeum delicate and thinner than female, with four longitudinal carinae, without oblique carinae (Fig. 2F). Outside of hind femora spots larger than female (Fig. 2A). Gaster with three segments visible. LT1: WT1 = 38: 62. Posterior of T1 and T2, most of T3 with tiny punctures, reminder smooth. S2 and S3 scattered with tiny punctures, but S3 denser, punctures on S3 converged longitudinally to form rugose towards apex, posterior margin significantly transverse, arched concave (Fig. 2A, H). Genitalia from above, penis valves at apex elongated oval, each half on inner ventral margin serrate, conspicuously short, laterally bulged and meeting in a midline; parameres broad at base, apical part broadly pale and frequently folded over, with few erect bristles at apex. Genitalia from beneath, each half of parameres scattered with short bristles; digitus and cuspis of volsella at apex fuscous, digitus produced vertically like a short, thick bill (Fig. 2I–K).

#### Distribution.

China (Guangdong, Jiangxi, Yunnan).

#### Etymology.

The specific name *albipedigerus* is derived from the Latin stem *alb* - (= white) and the Latin word *pedigerus* (= ped), which refers to the leg with ivory-white spots.

### ﻿ Dolichurus nigrilamellatus

Taxon classificationAnimaliaHymenopteraAmpulicidae

Bai & Li sp. nov.

F392E302-1E7E-5CF5-A1F9-7712DE6D2E7B

https://zoobank.org/4F6920D5-AC32-436A-BBE9-AC6F3F667568

Fig. 3A–I

#### Type material.

***Holotype***. China • ♀; Guangdong Province, Shaoguan City, Nanling Nature Reserve, Scale Frame; 24°54′45.81′′N, 113°2′33.64′′E; 844 m a.s.l.; 22.IX−13.X.2020, collected by Institute of Zoology, Guangdong Academy of Sciences; malaise trap. China • 1♀; Jiangxi Province, Ganzhou City, Jiulian Nature Reserve, Wudang Town, Chunhui Farm; 24°36′13.18′′N, 114°42′18.88′′E; 467 m a.s.l.; 7.VI.2021, collected by Yongying Ruan; light trap.

#### Diagnosis.

The new species is similar to *D. aposanus*Tsuneki et al., 1992, but differs from it and other congeners by the following characters (characters of *D. aposanus* in parentheses): 1) frontal line weak, frons with dense rugae converging toward midline above lamella (frontal line absent, frons sparsely punctate); 2) inner margin of mandible bidentate (inner margin of mandible tridentate); 3) A3/aW = 4, A12/bW = 4.2 (A3/aW = 4.5, A12/bW = 6.0); 4) eyes: gena = 29: 15 (eyes: gena = 34: 10); 5) sternaulus present (sternaulus absent); and 6) posterior margin of T1-T3 tiny puncture-rows absent medially (posterior margin of T1-T3 with complete tiny puncture-rows).

#### Description.

**Figure 3.**
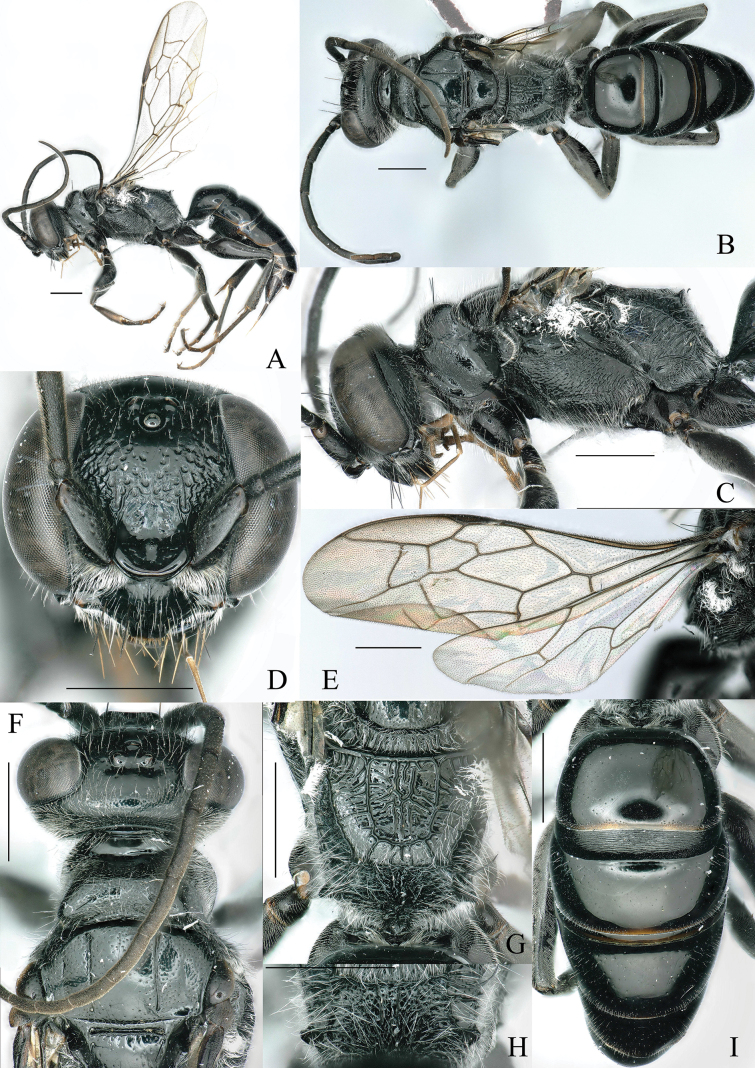
A–I. *Dolichurus nigrilamellatus* Bai & Li, sp. nov., holotype, ♀. A. Habitus, lateral view; B. Habitus, dorsal view; C. Head and thorax, lateral view; D. Head, frontal view; E. Wing; F. Head, pronotum, scutum, and scutellum, dorsal view; G. Scutellum, metanotum, and propodeum, dorsal view; H. Propodeum, posterior view; I. Gaster, dorsal view. Scale bars: 1 mm.

**Female**. Body length 12.3−12.9 mm. Black, apical margin of lamella dark brown. Mandible ferruginous-orange; maxillary and labial pale brown. Tegula dark brown. Inner surface of fore tibiae apically light yellowish brown, tarsi brown. Wing hyaline and iridescent, veins and stigma brown (Fig. 3E). Vestiture with silvery pubescence; most of frons and vertex with silvery erect pubescence; side of lamella and frons below lamella with appressed silvery pubescence, mandible with yellow erect macrochaetae. Gaster smooth, practically impunctate; S1 covered with dense silvery setae. Long black bristles on clypeus 4, on frons 4, on pronotum 2 and on scutellum 4 (Fig. 3A, B).

***Head***. In front view, nearly subcircular in outline, HW: HL = 97: 87. Mandible broad, acute apically, inner margin bidentate. Clypeus smooth, convex medially, conspicuous median carina reaching subapical region practically, apical lobe truncate, base width: middle width: apical width = 13: 50: 10 (Fig. 3D). Antennae 12 segments, scape with very weak ventral carina. Relative length of antennal segments I: II: III: IV: V: VI; VII: VIII: IX: X: XI: XII = 32: 7: 32: 32: 32: 30: 27: 25: 25: 23: 21: 21. A3/aW = 4.0, A12/bW = 4.2. Lamella parallel on both sides, apical margin thickened, length: width = 27: 25. Frontal line weak, frons with dense rugae converging toward midline, mixed with large punctures; remainder smooth. Inner margins of eyes slightly convergent above, IODv: IODc = 42: 51. POD: OD: OOD = 8: 4: 13, both side of ocelli with tiny punctures (Fig. 3D). In dorsal view, vertex shiny, scattered with tiny punctures, slightly arched above eyes (Fig. 3F). In lateral view, eyes: gena = 29: 15 (Fig. 3C).

***Thorax***. Pronotum smooth, scattered with tiny punctures, without transverse carina anteriorly, anteriorly densely punctate, pronotal tubercle slightly raised, hardly projects over level of scutum (in lateral view), median impression shallow, pronotal lobe with conspicuous rugae (Fig. 3C, F). Scutum and scutellum shiny, scattered with tiny punctures (Fig. 3F). Mesopleuron coarsely rugose, scrobal sulcus conspicuous, depressed, sternaulus consisting of rough large punctures (Fig. 3C). Metanotum with longitudinal rugae (Fig. 3G). Dorsal surface of propodeum with four longitudinal carinae, including two longitudinal carinae, and two oblique, longitudinal carinae located further out to sides (Fig. 3G). Posterior surface of propodeum with radiating reticulation, lateral margin with blunt tooth halfway from dorsum (Fig. 3H). In lateral view, dorsal surface and posterior surface forming obtuse angle, lateral surface of propodeum with oblique carinae (Fig. 3C). Dorsal surface of mid and hind tibiae with two rows of parallel spines, ventral surface of tarsi with two rows of parallel spines (Fig. 3A).

***Gaster***. Six segments visible. LT1: WT1 = 45: 76. Lateral side of T1 with conspicuous longitudinal carinae. Lateral margin of terga with few tiny punctures, S4 and apical margin of S5-S6 with tiny punctures, remainder smooth (Fig. 3A, I).

#### Distribution.

China (Guangdong, Jiangxi).

#### Etymology.

The specific name *nigrilamellatus* is derived from the Latin stem *nigr* - (= black) and the Latin word *lamellatus* (= lamella), which refers to the lamella being black in the female.

## ﻿Discussion

Cockroach wasps exhibit considerable species diversity and prey on cockroaches, which are widely distributed in various ecological habitats, such as cities, villages, and forests. Cockroach wasps play a crucial role in regulating cockroach population dynamics, serving as an important group of predatory natural enemies.

In this study, two new species were collected in Yunnan Province (Xishuangbanna Tropical Botanical Garden, rubber plantation and rainforest), Guangdong Province (Nanling Nature Reserve) and Guangxi Zhuang Autonomous Region (Jiulian Nature Reserve). The collection localities with high vegetation coverage and moderate temperature and humidity are suitable for hunting, growth and reproduction. *Dolichurus* search for prey near shallow ditches along steep riverbanks and piles of fallen leaves and withered foliage. During previous field investigations, we found that *Dolichurus* have limited distributions and a low individual density.

Furthermore, the discovery of two new species in this study expands the distribution of *Dolichurus* in the Oriental regions. Considering the limitations of the investigation, we speculate that there are still some species unrecognized in this genus and other tribes. Therefore, it is necessary to collect more materials for research in the future.

## Supplementary Material

XML Treatment for Dolichurus

XML Treatment for Dolichurus albipedigerus

XML Treatment for Dolichurus nigrilamellatus

## ﻿Additional information

### Use of AI

No use of AI was reported.

### Author ORCIDs

Xuemei Bai https://orcid.org/0009-0000-9779-6276

Li Ma https://orcid.org/0000-0002-3436-1387

Guowen Tang https://orcid.org/0009-0004-0627-0789

Qiang Li https://orcid.org/0000-0001-5950-8843

### Data availability

All of the data that support the findings of this study are available in the main text.
